# Disruption of beta cell acetyl-CoA carboxylase-1 in mice impairs insulin secretion and beta cell mass

**DOI:** 10.1007/s00125-018-4743-7

**Published:** 2018-10-17

**Authors:** James Cantley, Aimee Davenport, Laurène Vetterli, Nandor J. Nemes, P. Tess Whitworth, Ebru Boslem, Le May Thai, Natalie Mellett, Peter J. Meikle, Kyle L. Hoehn, David E. James, Trevor J. Biden

**Affiliations:** 1 Department of Physiology, Anatomy and Genetics, University of Oxford, Parks Road, Oxford, OX1 3PT UK; 2 Diabetes and Obesity Research Program, Garvan Institute of Medical Research, Darlinghurst, NSW Australia; 3 Baker IDI Heart and Diabetes Institute, Melbourne, VIC Australia; 4 School of Biotechnology and Biomolecular Sciences, University of New South Wales, Sydney, NSW Australia; 5 The Charles Perkins Centre, School of Molecular Biosciences, School of Medicine, University of Sydney, Sydney, NSW Australia; 6 St Vincent’s Clinical School, Faculty of Medicine, University of New South Wales, Darlinghurst, NSW Australia

**Keywords:** ACC1, Beta cell, Insulin secretion, Islet, Lipid metabolism, Mouse, mTOR

## Abstract

**Aims/hypothesis:**

Pancreatic beta cells secrete insulin to maintain glucose homeostasis, and beta cell failure is a hallmark of type 2 diabetes. Glucose triggers insulin secretion in beta cells via oxidative mitochondrial pathways. However, it also feeds mitochondrial anaplerotic pathways, driving citrate export and cytosolic malonyl-CoA production by the acetyl-CoA carboxylase 1 (ACC1) enzyme. This pathway has been proposed as an alternative glucose-sensing mechanism, supported mainly by in vitro data. Here, we sought to address the role of the beta cell ACC1-coupled pathway in insulin secretion and glucose homeostasis in vivo.

**Methods:**

*Acaca*, encoding ACC1 (the principal ACC isoform in islets), was deleted in beta cells of mice using the Cre/loxP system. *Acaca* floxed mice were crossed with *Ins2*cre mice (βACC1KO; life-long beta cell gene deletion) or *Pdx1*creER mice (tmx-βACC1KO; inducible gene deletion in adult beta cells). Beta cell function was assessed using in vivo metabolic physiology and ex vivo islet experiments. Beta cell mass was analysed using histological techniques.

**Results:**

βACC1KO and tmx-βACC1KO mice were glucose intolerant and had defective insulin secretion in vivo. Isolated islet studies identified impaired insulin secretion from beta cells, independent of changes in the abundance of neutral lipids previously implicated as amplification signals. Pancreatic morphometry unexpectedly revealed reduced beta cell size in βACC1KO mice but not in tmx-βACC1KO mice, with decreased levels of proteins involved in the mechanistic target of rapamycin kinase (mTOR)-dependent protein translation pathway underpinning this effect.

**Conclusions/interpretation:**

Our study demonstrates that the beta cell ACC1-coupled pathway is critical for insulin secretion in vivo and ex vivo and that it is indispensable for glucose homeostasis. We further reveal a role for ACC1 in controlling beta cell growth prior to adulthood.

**Electronic supplementary material:**

The online version of this article (10.1007/s00125-018-4743-7) contains peer-reviewed but unedited supplementary material, which is available to authorised users.

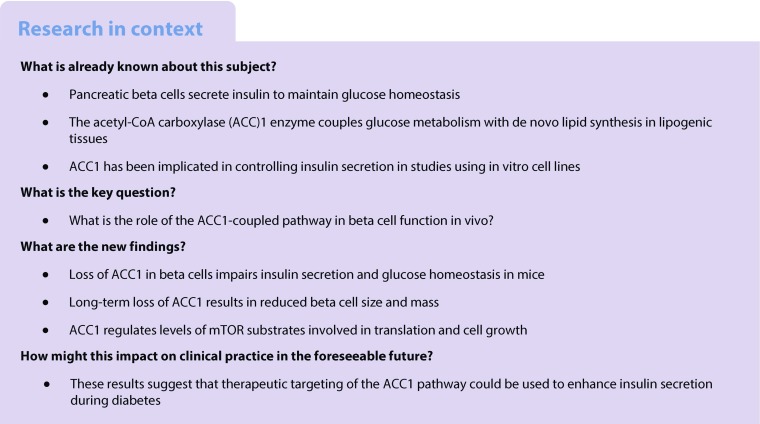



## Introduction

Beta cells adapt to metabolic challenges by increasing insulin secretory output to maintain glycaemic control. This occurs not only acutely in response to elevations in blood glucose but also over the longer term during obesity and pregnancy, via an expansion of beta cell mass and an enhancement of secretory function. Conversely, failure of beta cell output to match demand results in hyperglycaemia and progression towards type 2 diabetes. Delineating the mechanisms that maintain a functional beta cell mass is therefore a key goal for diabetes research. Surprisingly, there are still gaps in our understanding of acute stimulus–secretion coupling, longer term adaptations and the inter-relationships between these two processes, which are often studied independently.

Glucose stimulates insulin secretion by sequentially increasing glucose oxidation, ATP production, K_ATP_ channel closure and calcium influx [1]. Acting in concert with this triggering pathway, other glucose-regulated pathways enhance insulin secretion [2]. The factors underlying these amplification pathways remain poorly understood [3], although lipid metabolites may play a role [4]. Beta cells are dependent on glucose oxidation to trigger insulin secretion, although only ~60% of glucose enters mitochondria via pyruvate dehydrogenase (driving oxidation); the remaining ~40% of glucose-derived pyruvate enters mitochondria via pyruvate carboxylase [5], supporting an unexpectedly high rate of glucose-driven anaplerotic flux and cellular citrate accumulation [6], which closely tracks the insulin secretory response [7]. This leads to the concept of an alternative glucose-sensing mechanism utilising this anaplerotic flux.

Acetyl-CoA carboxylase (ACC) enzymes couple glucose and lipid fluxes through anabolic and catabolic pathways. ACC enzymes are allosterically activated by cytosolic citrate [8] to catalyse malonyl-CoA synthesis from acetyl-CoA (which can be derived from citrate). Malonyl-CoA is a substrate of fatty acid synthase (FAS) and an inhibitor of carnitine-palmitoyl transferase 1: therefore, ACC activity promotes lipid production and repression of fat oxidation [9]. Of the two ACC isoforms, ACC1 is expressed in lipogenic tissues such as hepatocytes and adipocytes, whereas ACC2 is expressed in oxidative tissues such as skeletal muscle [10] where it is localised to the mitochondrial surface and regulates β-oxidation [11]. Both malonyl-CoA and long-chain acyl-CoAs, generated downstream of ACC, have been proposed as metabolic coupling factors in beta cells [12–14]. ACC1 but not ACC2 is expressed in human [15] and rodent islets [15, 16].

In vitro evidence linking ACC1 to insulin secretion in beta cells includes the stimulation of ACC activity in response to glucose [17] and the impairment of glucose-stimulated insulin secretion (GSIS) following pharmacological or genetic inhibition of ACC or depletion of malonyl-CoA [15, 16, 18, 19]. However, the role of ACC1 in beta cell function and adaptation has not been examined in vivo and the physiological role of this pathway has yet to be determined. Global deletion of the gene encoding ACC1 (*Acaca*) is embryonically lethal [10]. Therefore, the aim of our present study was to generate mice with the *Acaca* gene deleted specifically in beta cells, using two temporally distinct genetic approaches, to assess the role of beta cell ACC1 activity in insulin secretion and glucose homeostasis in vivo.

## Methods

For detailed methods, please refer to the electronic supplementary material (ESM) Methods.

### Mouse models

To disrupt ACC1 activity in beta cells, *Acaca* floxed mice [20, 21] were crossed with *Ins2*cre mice (Tg(*Ins2*-cre)25Mgn) [22] to generate *Ins2*cre-*Acaca*^flox/flox^ (βACC1KO) and littermate control *Ins2*cre (INS2cre) mice. To enable tamoxifen-inducible disruption of ACC1 activity in beta cells, *Acaca* floxed mice were crossed with *Pdx1*creER mice (Tg(*Pdx1*-cre/Esr1*)35.10Dam) [23]: experimental mice received s. c. tamoxifen injections at 10 weeks of age to generate tamoxifen-treated *Acaca*^flox/flox^, *Pdx1*-creER (tmx-βACC1KO) and littermate control tamoxifen-treated *Acaca*^flox/flox^ (tmx-Control) mice, providing temporal resolution on the role of ACC1 in beta cell function. Mice were maintained on a C57Bl/6 J background.

### Metabolic testing

Glucose tolerance tests were performed after a 16 h fast, by i. p. (2 g/kg) or i. v. (1 g/kg) injection of glucose. Insulin action was assessed by i. p. injection of insulin (0.75 U/kg) following a 6 h fast. Blood was sampled from the tail. Percentage body fat was determined by dual energy x-ray absorptiometry (DEXA) scan normalised to body weight.

### Islet isolation, culture and insulin secretion assays

Mouse islet studies were performed as previously described [24]. The pancreas was perfused with collagenase solution via the common bile duct, before excision, digestion at 37°C and mechanical disruption. Islets were recovered using a Ficoll-Paque gradient (GE Healthcare, Chalfont St Giles, UK) and cultured overnight. To assess ex vivo insulin secretion, islets were pre-incubated for 1 h in HEPES-buffered KRB (KRBH) containing 0.1% BSA and 2 mmol/l d-glucose, before batches of five size-matched islets were incubated at 37°C for 1 h with glucose, palmitate-BSA [25], KCl or diazoxide.

### Hormone and DNA assays

Insulin was quantified by ELISA (Crystal Chem, Elk Grove Village, IL, USA) or RIA (Merck Millipore, Burlington, MS, USA). Glucagon was quantified by RIA (Merck Millipore). Total pancreatic hormone content was quantified following homogenisation in ice-cold acid–ethanol. Batches of islets were lysed and DNA content quantified by SYBR green assay (ThermoFisher, Waltham, MA, USA) using salmon-sperm DNA standards [25].

### Islet metabolic studies

Glucose tracer measurements were performed as described [26]. Batches of 100 islets were cultured for 2 h at 37°C in KRBH containing 0.1% BSA and 2.8 or 20 mmol/l D-glucose with 0.5 × 10^6^ or 0.07 × 10^6^ MBq/mol d-[U-^14^C]glucose, respectively; (Perkin Elmer, Waltham, MA, USA). To quantify tracer oxidation, media was acidified and ^14^CO_2_ trapped via reaction with 0.1 ml KOH before liquid scintillation spectrometry. To quantify tracer incorporation into cellular lipids, a chloroform–methanol (2:1 vol./vol.) extraction was performed and fractions assayed by scintillation spectrometry.

MS based lipidomic analysis of islets was performed as described [27]. Lipids were extracted from 100 islets per mouse using chloroform–methanol (2:1) with a panel of internal lipid standards. Analysis was performed by electrospray ionisation–tandem MS using an API 4000 Q/TRAP mass spectrometer (Sciex, Framingham, MA, USA) with a turbo-ionspray source and Analyst 1.5 data system, following prior liquid chromatographic separation. Quantification of individual lipid species was performed using scheduled multiple-reaction monitoring in positive ion mode. Lipid concentrations were calculated by relating the peak area of each species to the peak area of the corresponding internal standard.

### Histology

Formalin-fixed paraffin-embedded pancreases were sectioned and immunostained using the primary antibodies indicated. For morphometry, stained sections were imaged with an Arperio slide scanner (Leica Biosystems, Wetzlar, Germany), and analysis performed using Imagescope software (Leica Biosystems): beta cell mass was calculated as described [28] using data averaged from three pancreatic sections per mouse. For dual immunofluorescent staining, sections were imaged using a DMI6000 SP8 Confocal microscope (Leica Biosystems).

### Western blotting

Tissue and cells were lysed in radioimmunoassay precipitation (RIPA) buffer and western blotting performed as previously described [24] using the primary antibodies indicated. Band density was quantified using Image J software (NIH, Bethesda, MA, USA). ACC1 protein was identified by avidin–agarose bead pull-down, SDS-PAGE, transfer to polyvinylidene difluoride (PVDF), incubation with streptavidin–horseradish peroxidase (HRP) (ThermoFisher) and chemiluminescent detection: this method utilises the affinity of the endogenous ACC1-bound biotin for avidin/streptavidin and has been described previously [20].

### Statistics

Data are presented as mean ± SEM. Simple pairwise comparisons were made using two-tailed *t* tests (unpaired unless otherwise stated). Multiple comparisons were made using two-way ANOVA with Bonferroni post-tests. A *p* value <0.05 was regarded as statistically significant. Statistics were performed using Prism6 (Graphpad Software, San Diego, CA, USA).

## Results

### Loss of ACC1 in *Ins2*-expressing tissues blocks glucose-driven de novo lipogenesis in beta cells

**Fig. 1:**
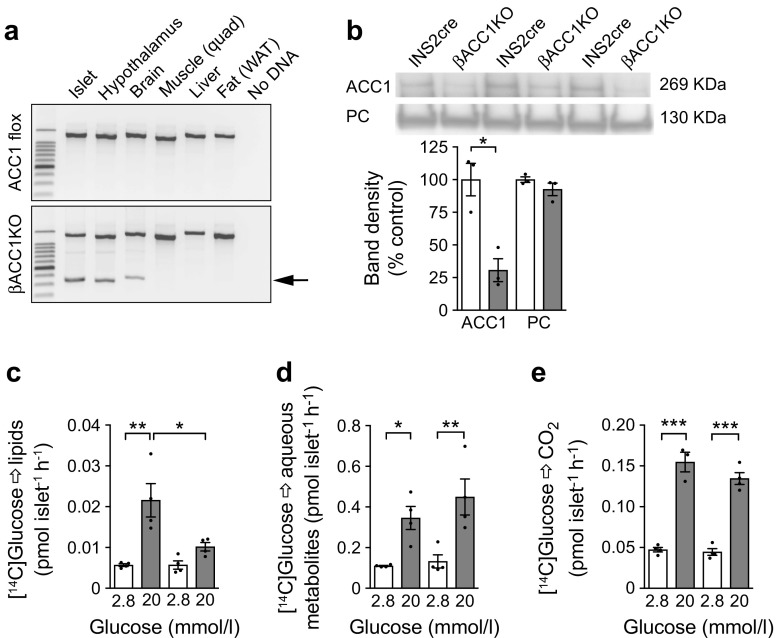
Deletion of *Acaca* in beta cells impairs glucose-driven lipogenesis in mice. (**a**) PCR analysis to confirm deletion of the floxed *Acaca* allele (indicated by arrow) in *Ins2*cre-*Acaca*^flox/flox^ (βACC1KO) but not in *Acaca*^flox/flox^ (ACC1 flox) mice. A control PCR confirmed amplifiable DNA template in all samples (upper band). (**b**) ACC1 protein in islet lysates assessed by avidin pull-down and streptavidin–HRP detection. Pyruvate carboxylase (PC) was used as a loading control. *n*=3 batches of islets from independent mice. (**c**–**e**) A d-[U-^14^C]glucose tracer was used to measure glucose-driven metabolic flux in islets isolated from mice and incorporation into cellular lipid pools (**c**), aqueous metabolites (**d**) and glucose oxidation (**e**) were quantified. White bars, INS2cre mouse islets; grey bars, βACC1KO mouse islets. *n*=4 batches of islets from independent mice. Data are presented as mean ± SEM. *p<0.05, **p<0.01 and ***p<0.001 (unpaired two-tailed *t* test). Quad, quadriceps; WAT, white adipose tissue

To test the role of the ACC1-coupled pathway in beta cells we generated mice with beta cell *Acaca* deletion (βACC1KO) in *Ins2*-expressing tissues. PCR of genomic DNA revealed recombination of loxP sites only in the islets, hypothalamus and brain of βACC1KO mice, and not in the other tissues tested; recombination could not be detected in any of the tissues tested in *Acaca* floxed mice in the absence of Cre-recombinase (Fig. 1a). ACC1 protein levels were reduced by 70% in βACC1KO mouse islets (Fig. 1b), consistent with the >95% beta cell gene deletion efficiency reported for the INS2cre mouse [29] and the 75% beta cell content of mouse islets [30]. ACC1 function was ablated in βACC1KO mouse islets, as determined by the impaired flux of d-[U-^14^C]glucose tracer into total lipid pools (Fig. 1c). In contrast, flux into aqueous metabolites (Fig. 1d) and glucose oxidation (Fig. 1e) were unaltered. The residual ACC1-insensitive flux of d-[U-^14^C]glucose into βACC1KO mouse islet lipids may reflect de novo lipogenesis in alpha cells and/or incorporation of glycerol-3-phosphate, derived from glycolysis, into glycerolipids [31].

Pre-labelling of islets with [U-^14^C]palmitate revealed that incorporation of exogenous fatty acids into beta cell lipid pools or aqueous metabolites is not affected by loss of ACC1 (ESM Fig. 1a,b). Moreover, the rate of β-oxidation was very low in islets relative to glucose oxidation and was not altered by ACC1 loss (ESM Fig. 1c), consistent with ACC1 (the principal ACC isoform in islets [15, 16]) catalysing malonyl-CoA synthesis for lipogenesis rather than inhibition of β-oxidation.

To assess how these alterations in flux might impact on lipid mass, we undertook mass spectroscopic lipidomics. This revealed that loss of ACC1 did not alter the abundance of major neutral lipid species of diacylglycerol (DAG) and triacylglycerol (TAG) (ESM Fig. 1d,e), including key glucose-sensitive species [32]. In contrast, some forms of phospholipid, especially high-abundance species such as 34:1 phosphatidylcholine and 38:4 phosphatidylinositol, were significantly diminished (ESM Fig. 1f–i). This suggests that ACC1-driven de novo fatty acid synthesis plays little role in maintaining neutral lipid pools in the beta cell but makes a small but significant contribution to the abundance of some major phospholipids.

### Loss of ACC1 in *Ins2*-expressing tissues impairs insulin secretion and glucose homeostasis in vivo

**Fig. 2:**
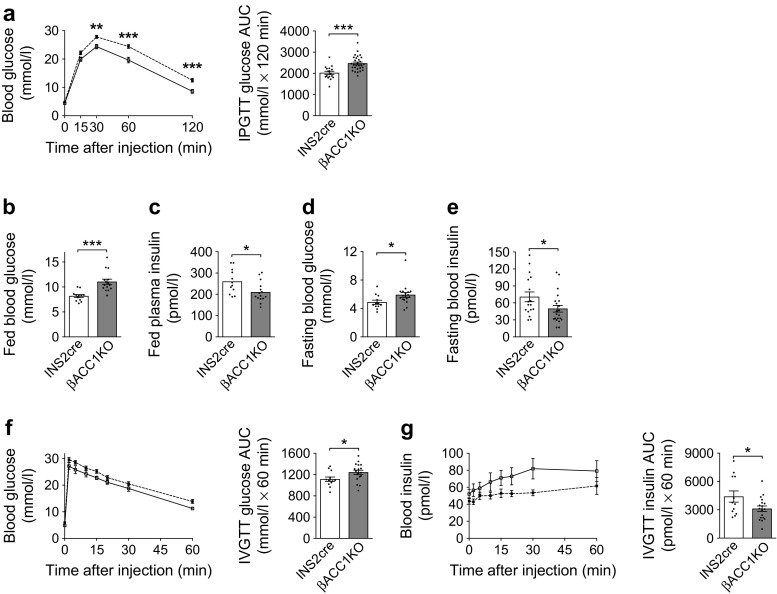
Loss of ACC1 in beta cells causes impaired glucose homeostasis and defective insulin secretion in vivo. (**a**) Blood glucose levels and AUC during an IPGTT (16 h fast; 2 g/kg glucose dose) performed in 12-week-old male βACC1KO mice (*n*=29) and INS2cre mice (*n*=17); *p*<0.001 for genotype effect across all time points (two-way ANOVA). (**b**–**e**) Blood glucose and circulating insulin in 12-week-old mice in the fed state (**b**, **c**; *n*=12 INS2cre mice and 15 βACC1KO mice) or after 16 h fasting (**d**, **e**; *n*=17 INS2cre mice and 21 βACC1KO mice). (**f**) Blood glucose levels and AUC during an IVGTT (16 h fast; 1 g/kg glucose dose) performed in 12-week-old male βACC1KO mice (*n*=18) and INS2cre mice (*n*=12); *p*=0.0304 for genotype effect (two-way ANOVA). (**g**) GSIS was assessed during the IVGTT. White squares and bars, INS2cre mice; black squares and grey bars, βACC1KO mice. Data are presented as mean ± SEM. *p<0.05, **p<0.01 and ***p<0.001 (unpaired two-tailed *t* tests for bar charts; two-way ANOVA with Bonferroni multiple comparisons test for time plots, for βACC1KO vs INS2cre at time point shown)

To investigate the role of ACC1 in beta cell function in vivo, we assessed glucose homeostasis in βACC1KO mice at 12 weeks of age (young adult). Intraperitoneal glucose tolerance tests (IPGTTs) revealed significantly elevated blood glucose levels in βACC1KO mice, relative to INS2cre control mice (Fig. 2a). In the fed and (16 h) fasted state, βACC1KO mice displayed significant elevations of blood glucose and corresponding decreases in circulating insulin (Fig. 2b–e): these data suggest that insufficient insulin secretion underlies the glucose-intolerant phenotype. This was further supported by an IVGTT, which confirmed glucose intolerance (Fig. 2f), along with a reduction in basal and GSIS (Fig. 2g). This clearly demonstrates that ACC1 controls insulin secretion in vivo.

### Loss of ACC1 in *Ins2*-expressing tissues does not alter adiposity or insulin action

**Fig. 3:**
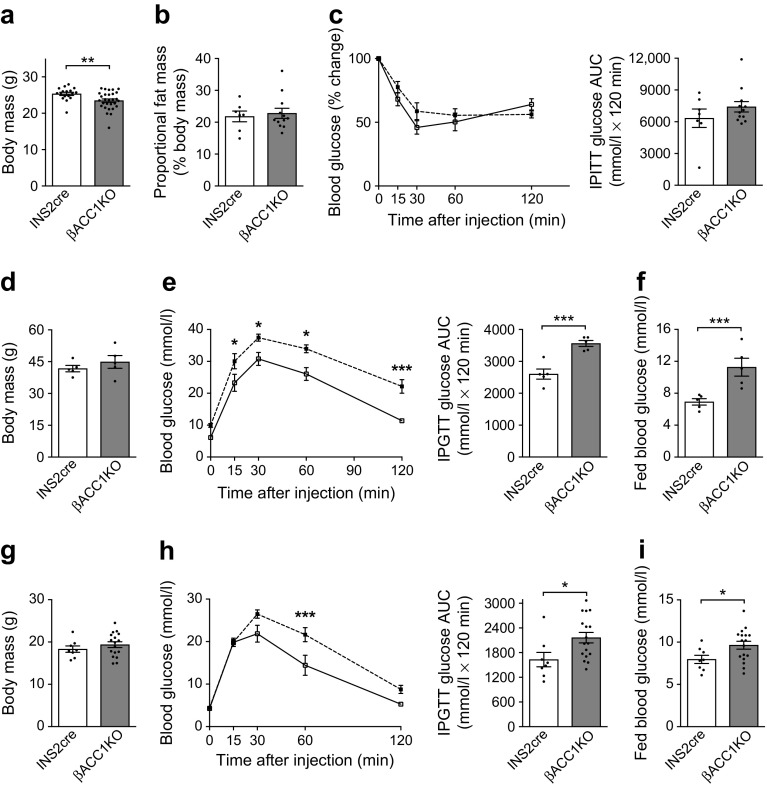
Insulin action, adiposity and body weight do not underpin glucose intolerance in βACC1KO mice. (**a**–**c**) Body weight at 12 weeks of age (**a**, *n*=17 INS2cre mice and 29 βACC1KO mice), proportional body fat (by DEXA scan) at 14 weeks of age (**b**, *n*=7 INS2cre mice and 12 βACC1KO mice) and blood glucose levels and AUC during an IPGTT (6 h fast; 0.75 U/kg insulin dose) at 15 weeks of age (**c**, *n*= 7 INS2cre mice and 12 βACC1KO mice) in male mice. (**d**–**f**) Body weight (**d**), blood glucose levels and AUC during an IPGTT (16 h fast, 2 g/kg glucose dose) (**e**) and fed blood glucose (**f**) in 12-month-old male mice. *n*=5 INS2cre and 5 βACC1KO; *p*=0.0005 for genotype effect across all time points (two-way ANOVA) in (**e**). (**g**–**i**) Body weight (**g**), blood glucose levels and AUC during an IPGTT (16 h fast, 2 g/kg glucose dose) (**h**) and fed blood glucose levels (**i**) in 12-week-old female mice; *n*=8 INS2cre mice and 17 βACC1KO mice; *p*=0.0337 for genotype effect across all time points (two-way ANOVA) in (**h**). White bars and squares, INS2cre mice; grey bars and black squares, βACC1KO mice. Data are presented as mean ± SEM. *p<0.05, **p<0.01 and ***p<0.001 (unpaired two-tailed *t* tests for bar charts; two-way ANOVA with Bonferroni multiple comparisons test for time plots, for βACC1KO vs INS2cre at time point shown)

The INS2cre mouse line can promote gene deletion in neuronal populations [33], with associated metabolic phenotypes such as weight gain and hyperphagia [34]. Moreover, it has been reported that deletion of the gene encoding FAS, using the INS2cre mouse, results in changes in energy balance due to alterations in central peroxisome proliferator-activated receptor α (PPARα) signalling [35]. We therefore addressed neuronal metabolic signalling. Relative to INS2cre control mice, our βACC1KO mice showed a small but significant decrease in body weight at 12 weeks of age (Fig. 3a). This decrease was less pronounced than that reported for FAS deletion in INS2cre neurones [35]. However, we found no difference in proportional body fat (Fig. 3b) or insulin action (Fig. 3c) between the βACC1KO and INS2cre mice at 12 weeks of age, indicating that regulation of adiposity and insulin sensitivity is not affected by loss of ACC1 activity in INS2cre-expressing tissues. The small reduction in body weight in 12-week-old βACC1KO mice is unlikely to explain the impairment in glucose tolerance we observe because by 12 months of age βACC1KO mice had regained a normal body weight (Fig. 3d) yet remained profoundly glucose intolerant (Fig. 3e,f). Furthermore, 12-week-old female βACC1KO mice exhibited normal body weight (Fig. 3g) while displaying impaired glucose tolerance (Fig. 3h,i).

### ACC1 is necessary for normal insulin secretion ex vivo

**Fig. 4:**
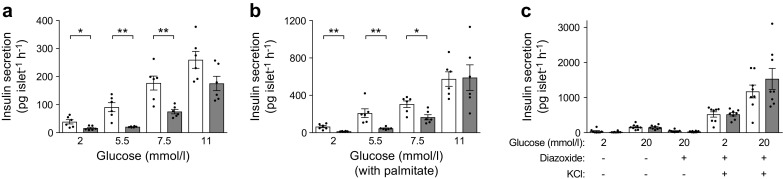
Impaired insulin secretion in islets isolated from βACC1KO mice. (**a**, **b**) Ex vivo insulin secretion assays were performed, using islets isolated from βACC1KO and INS2cre mice, in the absence (**a**) or presence (**b**) of 0.4 mmol/l palmitate (coupled to 0.92% BSA) to potentiate GSIS. Data were generated simultaneously in islets isolated from the same mice. (**c**) The amplifying pathways of GSIS were exposed by addition of diazoxide (100 μmol/l) and KCl (25 mmol/l) to islets. White bars, INS2cre mouse islets; grey bars, βACC1KO mouse islets. Data are presented as mean ± SEM, *n*=6 (**a**, **b**) or 8 (**c**) mice. *p<0.05 and **p<0.01 (unpaired two-tailed *t* test)

Assessment of islet function ex vivo revealed that insulin secretion was defective both at baseline and in response to glucose stimulation in βACC1KO mouse islets (Fig. 4a). This is consistent with previous reports that inhibition of the ACC1-coupled pathway disrupts glucose-stimulated [15, 16, 19] and basal [19] insulin secretion. Despite a marked reduction in the quantity of insulin secreted, βACC1KO islets retained some glucose responsiveness at 5.5 mmol/l glucose, relative to 2 mmol/l glucose (INS2cre 2.34-fold basal vs βACC1KO 1.28-fold basal), and this recovered further at higher glucose concentrations.

We next considered whether the defective insulin secretion in βACC1KO islets could be restored by provision of exogenous NEFA, which modulate GSIS by both intracellular and extracellular mechanisms [36]. Acute treatment of isolated islets with palmitate potentiated GSIS in both genotypes (Fig. 4b), demonstrating that the response to NEFA stimulation was intact. However, insulin release remained significantly impaired in βACC1KO mouse islets at basal and physiological glucose concentrations, in the presence of palmitate, relative to control mouse islets (Fig. 4b). Thus, the secretory defect in βACC1KO islets cannot be rescued with exogenous fatty acids.

Production of malonyl-CoA and modulation of cellular lipid levels has been implicated in the amplifying pathways of GSIS [4]. We therefore assessed these pathways using an established pharmacological method: the K_ATP_ channel is maintained open with diazoxide and the beta cell is artificially depolarised with KCl to induce calcium influx [2]. Under these conditions, marked amplification of insulin secretion by glucose was retained in both control and βACC1KO mouse islets (Fig. 4c). Therefore, ACC1 is not required for a functional amplifying pathway at high glucose concentrations.

Although loss of ACC1 activity inhibited insulin secretion at baseline and in response to physiological concentrations of glucose (Fig. 4a), no impairment was observed in response either to supraphysiological concentrations of glucose (20 mmol/l) or to KCl (Fig. 4c). This indicates that βACC1KO mouse islets have functional insulin exocytosis pathways but fail to respond to physiological glucose concentrations, suggesting a right shift in the GSIS curve. One explanation for this could be a reduction in glucokinase levels or mitochondria; however, levels of glucokinase and mitochondrial heat shock protein 9 (HSPA9) were unaltered in βACC1KO mouse islets (ESM Fig. 2a).

### ACC1 is required for beta cell growth

**Fig. 5:**
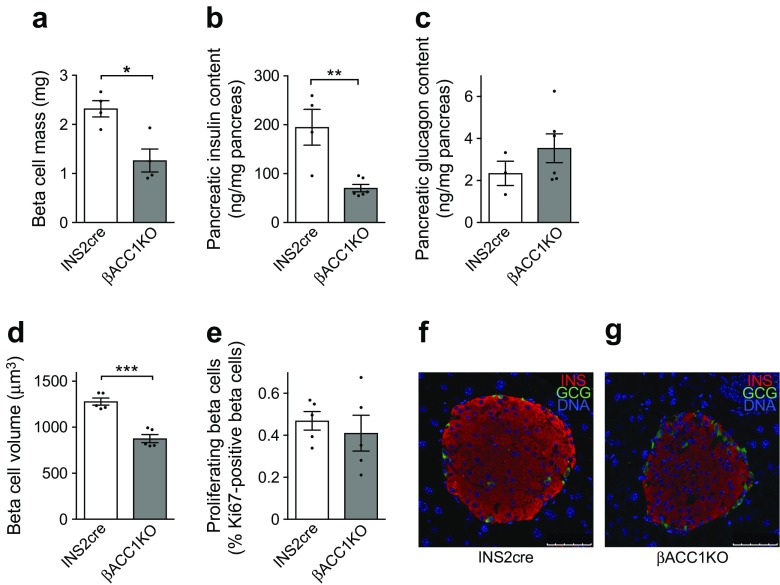
ACC1 is necessary for an adequate beta cell mass. (**a**) Morphometric analysis of immunostained pancreatic sections was performed to quantify beta cell mass. (**b**, **c**) Insulin (**b**) and glucagon (**c**) content of pancreases homogenised in acid–ethanol was determined by radioimmunoassay. (**d**) Further morphometric analysis revealed a reduction in mean beta cell volume, which was calculated by dividing insulin-positive area (containing at least 1000 cells) by the number of cells/nuclei in that area. (**e**) Pancreatic sections were immunostained for Ki67 and insulin to quantify beta cell proliferation rates. (**f**, **g**) Dual immunofluorescent imaging of formalin-fixed paraffin-embedded pancreatic sections for insulin (INS; red) and glucagon (GCG; green); scale bars, 50 μm. White bars, INS2cre mouse pancreas; grey bars, βACC1KO mouse pancreas. Data are presented as mean ± SEM. Each data point represents an individual mouse. *p<0.05, **p<0.01 and ***p<0.001 (unpaired two-tailed *t* test)

Using histological morphometric techniques, we next identified a significant 46% reduction in beta cell mass in βACC1KO mice, relative to INS2cre control mice (Fig. 5a), with a corresponding decrease in pancreatic insulin content (Fig. 5b). In contrast, glucagon content was not altered by genotype (Fig. 5c), arguing against a major phenotype in alpha cells. Interestingly, the observed decrease in beta cell mass appears to be partly explained by a 31% reduction in individual beta cell size (Fig. 5d), whereas both proliferation (Fig. 5e) and apoptosis (ESM Fig. 2b) rates were unaltered. To evaluate this further we measured DNA content as a surrogate of cell number, using equal numbers of size-matched islets: those from βACC1KO mice showed an 18% increase in DNA content relative to those from INS2cre mice (Table 1), indicating that these islets contain a greater number of smaller beta cells. In contrast, mean islet insulin content was not significantly altered in βACC1KO mice, except when normalised for islet DNA content (effectively normalising for cell number) (Table 1), reflecting reduced beta cell size. Dual immunofluorescent staining for insulin and glucagon in βACC1KO pancreatic sections showed normal islet architecture with a clear alpha cell mantle surrounding a beta cell core (Fig. 5f,g). These results reveal that ACC1 activity is necessary for the regulation of individual beta cell size and the overall mass of beta cells in the pancreas.

**Table 1:** DNA and insulin content of βACC1KO and INS2cre mouse islets

Genotype	Islet DNA content (ng)	Islet insulin content/DNA content (ng/ng)
INS2cre	14.67 ± 0.67	4.32 ± 0.49
βACC1KO	17.3 ± 1.03*	2.63 ± 0.54*

Data are presented as mean ± SEM, *n*=10 mice per genotype

Batches of 100 size-matched isolated islets per mouse were collected, lysed and DNA and insulin content quantified before normalising for islet number

*p<0.05 for βACC1KO vs INS2cre (unpaired two-tailed *t* test)

### Short-term loss of beta cell ACC1 activity in adult mice impairs glucose tolerance and insulin secretion in vivo

**Fig. 6:**
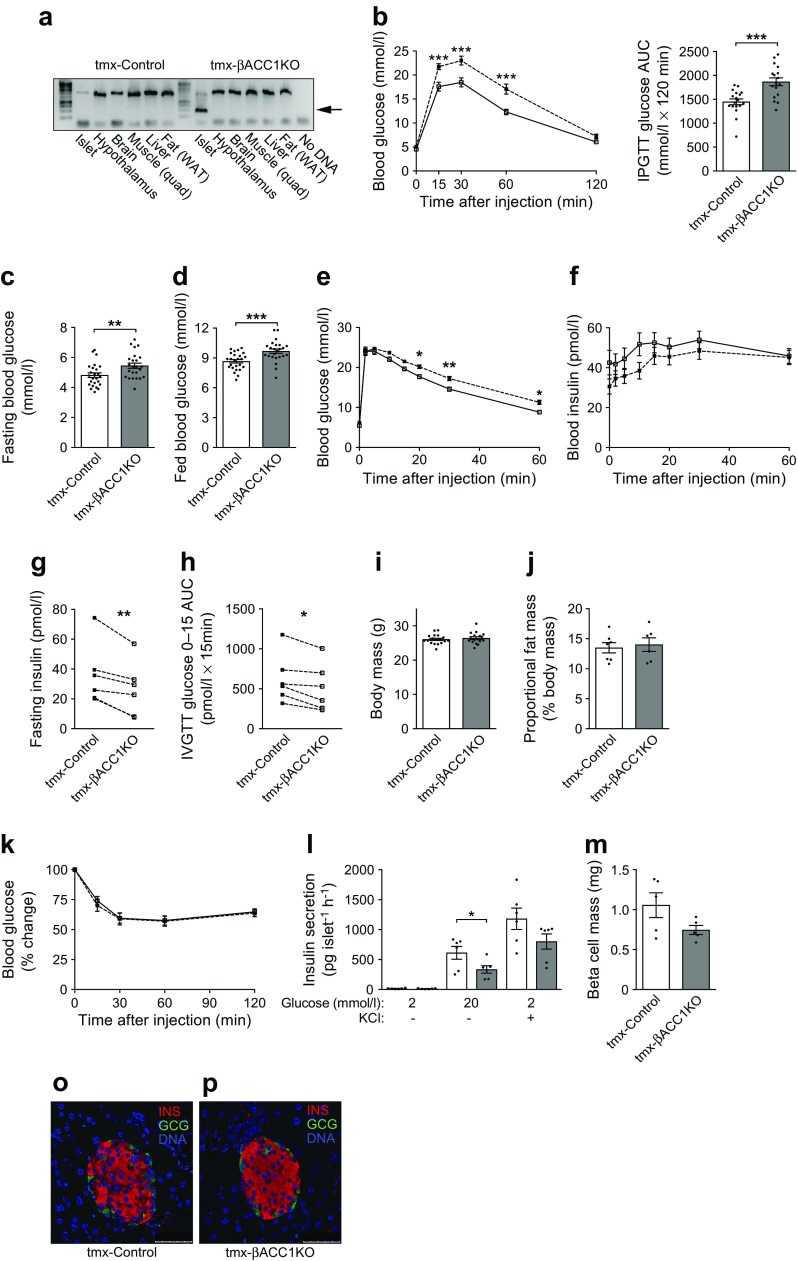
Short-term loss of beta cell ACC1 activity in adult mice causes glucose intolerance and impaired insulin secretion. Male mice received 2 × daily 8 mg tamoxifen s. c. injections at 10 weeks of age to generate tmx-βACC1KO and tmx-Control mice. (**a**) PCR analysis to confirm deletion of the floxed *Acaca* allele (lower band; indicated by arrow) 1 week post tamoxifen treatment in *Pdx1*creER-*Acaca*^flox/flox^ (tmx-βACC1KO) mice but not in *Acaca*^flox/flox^ (tmx-Control) mice. A control PCR confirmed amplifiable DNA template in all samples (upper band). (**b**) Blood glucose levels and AUC during an IPGTT (16 h fast, 2 g/kg glucose dose) in male tmx-βACC1KO mice (*n*=17) and control mice (*n*=18) at 14 weeks of age; *p*=0.0002 for genotype effect across all time points (by two-way ANOVA). (**c**, **d**) Blood glucose levels in 16 h fasted (**c**) and fed (**d**) 14-week-old male mice (*n*=24 tmx-βACC1KO mice or 25 tmx-Control mice). (**e**–**h**) IVGTT was carried out with 17-week-old male mice to assess GSIS in vivo (16 h fast, 1 g/kg glucose dose). Blood glucose and insulin levels (**e**, **f**, *n*=25 tmx-βACC1KO mice or 26 tmx-Control mice) were determined; *p*=0.0002 and *p*=0.0014 for genotype effect on glucose and insulin across all time points, respectively (two-way ANOVA). Fasted (16 h) blood insulin (**g**) and 0–15 min insulin AUC (**h**) were determined from the IVGTT data and presented as mean data from five independent cohorts of mice. (**i**–**k**) Body weight (**i**, *n*=17 tmx-βACC1KO mice and 18 tmx-Control mice), proportional body fat (**j**, *n*=6 tmx-βACC1KO mice and 7 tmx-Control mice) and intraperitoneal insulin action (6 h fast, 0.75 U/kg insulin i.p.) (**k**, *n*=17 tmx-βACC1KO mice and 18 tmx-Control mice) were measured when mice were aged 14 weeks (**i**, **j**) or 15 weeks (**k**). (**l**) Ex vivo insulin secretion was assayed using islets isolated from 15-week-old mice (*n*=6 per genotype). (**m**) Beta cell mass was quantified by morphometric analysis of immunostained pancreatic sections (*n*=5 per genotype). (**o**, **p**) Dual immunofluorescent imaging of formalin-fixed paraffin-embedded pancreatic sections from tmx-Control (**o**) and tmx-βACC1KO (**p**) mice stained for insulin (INS; red) and glucagon (GCG; green); scale bars, 50 μm. White bars and squares, tmx-Control mice or islets; grey bars and black squares, tmx-βACC1KO mice or islets. Data are presented as mean ± SEM. *p<0.05, **p<0.01 and ***p<0.001 (two-way ANOVA with Bonferroni multiple comparisons test for time plots, for tmx-βACC1KO vs tmx-Control at time point shown; unpaired two-tailed *t* tests for bar charts, except for **g** and **h**, which are paired by cohort). Quad, quadriceps; WAT, white adipose tissue

As the *Ins2* promoter is first activated around embryonic day 11.5 [37], we cannot discriminate between the role of ACC1 in beta cell development and its role in mature beta cell function using our βACC1KO model. To assess the role of ACC1 specifically in beta cells in adult mice, we crossed *Acaca* floxed mice with the tamoxifen-inducible *Pdx1*creER line [23]. Young adult male mice with inducible beta cell *Acaca* deletion (tmx-βACC1KO) were generated by injection of tamoxifen at 10 weeks of age; tamoxifen-treated *Acaca* floxed mice were used as a control group (tmx-Control). Specific deletion of the *Acaca* floxed allele was confirmed in islets isolated from tmx-βACC1KO but not tmx-Control mice (Fig. 6a). At 4 weeks post-tamoxifen administration (14 weeks of age), IPGTTs revealed pronounced glucose intolerance (Fig. 6b), accompanied by elevated fasted and fed blood glucose levels (Fig. 6c,d), in tmx-βACC1KO mice. During an IVGTT, tmx-βACC1KO mice showed glucose intolerance (Fig. 6e) with defective insulin secretion at baseline and in response to glucose stimulation (Fig. 6f–h). These data indicate that defective insulin secretion and elevated blood glucose levels due to loss of beta cell ACC1 activity are not due to developmental effects.

We are unaware of any reports of *Pdx1*creER transgene expression in the brain; however, a non-inducible *Pdx1*cre made by the same laboratory [23] showed Cre-recombinase activity in the hypothalamus and brainstem [33]. Notwithstanding this, we could not detect recombination of the *Acaca* floxed allele by PCR in the hypothalamus or brain of tmx-βACC1KO mice (Fig. 6a), whereas recombination was readily detectable in these tissues from βACC1KO mice (Fig. 1a). Furthermore, tmx-βACC1KO mice showed no evidence of a neuronal or insulin-resistant phenotype: body weight (Fig. 6i), adiposity (Fig. 6j) and insulin action (Fig. 6k) were all unaltered relative to control mice. Taken together, these data clearly indicate a beta cell specific phenotype in tmx-βACC1KO mice.

### Short-term loss of beta cell ACC1 activity in adult mice impairs insulin secretion, but not beta cell mass

We next isolated islets from tmx-βACC1KO mice at 6 weeks post-tamoxifen injection and assessed insulin secretion profiles. Interestingly, loss of ACC1 in this model impaired GSIS at 20 mmol/l glucose (Fig. 6l). This is in contrast with βACC1KO mouse islets, where secretion was defective at 2, 5.5 and 7.5 mmol/l glucose but not at 20 mmol/l glucose. The tmx-βACC1KO mouse islets did not show a significant difference in insulin secretion either at basal glucose or in response to KCl stimulation (Fig. 6l). Therefore, short-term loss of ACC1 activity in adult beta cells (tmx-βACC1KO) results in a pronounced defect in GSIS. In contrast, life-long loss of beta cell ACC1 activity (βACC1KO) results in defective insulin secretion at baseline and in response to moderate physiological glucose concentrations. Glucokinase and HSPA9 levels were unaltered in tmx-βACC1KO mouse islets, similar to βACC1KO islets (ESM Fig. 2c).

Pancreatic morphometry conducted with the tmx-βACC1KO model 6 weeks post-tamoxifen did not reveal any significant change in beta cell mass (Fig. 6m). Furthermore, DNA content from size-matched-islet preparations was not altered in tmx-βACC1KO mice relative to control mice (ng DNA/islet, mean ± SEM: tmx-Control 10.98 ± 0.65; tmx-βACC1KO 10.90 ± 0.99; *n* = 6 mice). Dual immunofluorescent staining for insulin and glucagon showed normal islet architecture in tmx-βACC1KO pancreatic sections (Fig. 6o,p). Taken together, these results suggest that beta cell size and mass are not altered following short-term loss of ACC1 activity in adult beta cells.

### mTOR substrate levels are reduced in βACC1KO mouse islets

**Fig. 7:**
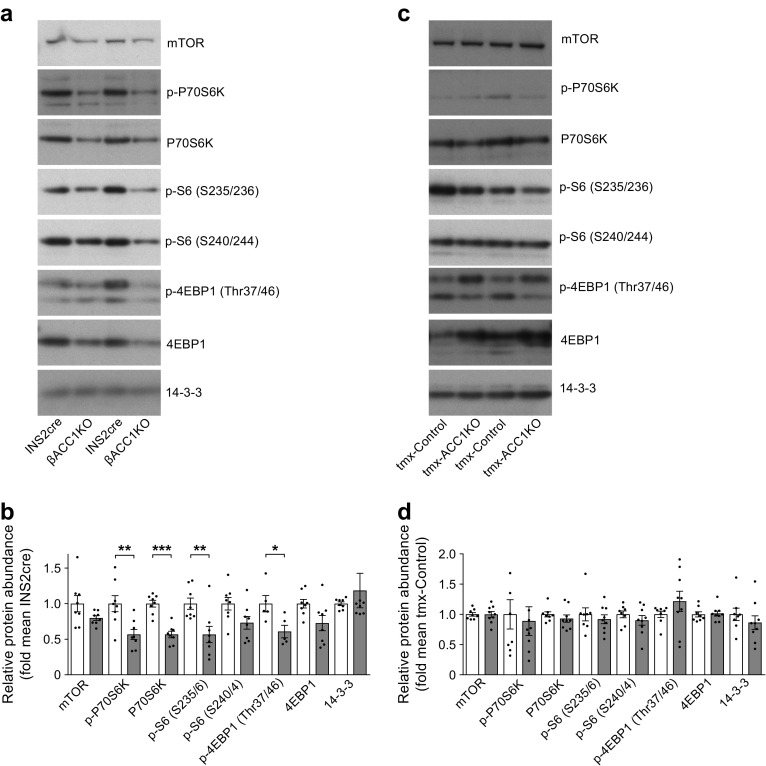
Chronic loss of beta cell ACC1 activity results in reduced levels of translational regulator proteins. Islets were isolated from 14-week-old male βACC1KO and INS2cre mice (**a**, **b**, *n*=8 mice per group) and from tmx-βACC1KO (*n*=9) and tmx-Control mice (*n*=8) 4 weeks post-tamoxifen injection (**c**, **d**). Western blotting analysis of islet lysates was performed using antibodies against the indicated proteins/phosphoproteins (**a**, **c**); quantification of band densities shows protein levels in each sample relative to the mean control signal (**b**, **d**). In (**b**), white bars, INS2cre mouse islets; grey bars, βACC1KO mouse islets. In (**d**), white bars, tmx-Control mouse islets; grey bars, tmx-βACC1KO mouse islets. Data are presented as mean ± SEM. *p<0.05, **p<0.01 and ***p<0.001 (unpaired two-tailed *t* test)

Our data reveal that long-term loss of ACC1 activity impairs insulin secretion and beta cell mass. Mechanistic target of rapamycin kinase (mTOR) is an anabolic serine/threonine kinase that controls protein biosynthesis, cell growth and cell proliferation [38]. Genetic activation of mTOR results in enhanced beta cell growth and increased insulin secretion in mouse beta cells [39], whereas pharmacological inhibition of mTOR dramatically inhibited insulin secretion and reduced beta cell mass in a rodent model of obesity [40]. Because mTOR is activated by de novo synthesis of phosphatidic acid [41], we hypothesised that loss of mTOR activity may underlie the reduction in beta cell size observed in βACC1KO islets. To investigate this, we performed western blotting for the mTOR kinase substrates responsible for translational control: ribosomal protein S6 kinase, polypeptide 1 (P70S6K) and eukaryotic translation initiation factor 4E binding protein 1 (4EBP1). Although there was a significant decrease in phosphorylation of these mTOR targets in βACC1KO mouse islets, consistent with a reduction in mTOR activity, this was accompanied by a proportional decrease in total protein levels of P70S6K (Fig. 7a,b). This resulted in reduced phosphorylation of the P70S6K substrate ribosomal protein S6 (Fig. 7a,b). In contrast, levels of these proteins/phosphoproteins were not altered in tmx-βACC1KO mouse islets, 6 weeks post-tamoxifen injection (Fig. 7c,d), consistent with the normal beta cell mass in this short-term *Acaca* deletion model. These data suggest that chronic loss of ACC1-coupled signalling in beta cells results in reduced activity of translational control proteins, which may underlie the reduced beta cell size in the βACC1KO model.

## Discussion

Our study demonstrates for the first time that ACC1 activity is required to maintain a functional beta cell mass and glucose homeostasis in vivo, thereby extending prior in vitro findings [15–19]. Using both acute and chronic beta cell gene deletion models, we show that the ACC1 pathway is necessary for adequate insulin secretion in vivo*.* This work identifies a novel role for the ACC1 pathway in regulating beta cell size that is temporally distinct from its effects on beta cell function. The ACC1 protein is present in human islets [15], indicating that our results are relevant for understanding human beta cell function.

Our study made use of two independent murine Cre lines, enabling us to distinguish the life-long effects of loss of beta cell ACC1 activity (βACC1KO mouse) from the adult-specific effects (tmx-βACC1KO mouse). Both models demonstrate that beta cell ACC1 plays a critical role in insulin secretion in vivo and ex vivo. Interestingly, there was a pronounced defect in GSIS at 20 mmol/l glucose in tmx-βACC1KO mice, consistent with the impaired secretion in response to glucose concentrations >16 mmol/l observed using pharmacological ACC1 inhibition in vitro [15, 16, 18, 19]*.* In contrast, the secretory defect in βACC1KO mice was only present at mid-physiological and basal glucose concentrations, suggestive of a degree of compensation to restore insulin secretion at higher glucose concentrations following long-term loss of beta cell ACC1. Furthermore, impairments in basal insulin secretion are consistent with reports using malonyl-CoA depleted INS(832/13) beta cells [19].

Although it is clear from our data and those of others that ACC1 activity is required for normal insulin secretion, the mechanism(s) underlying this effect have been unclear. One early proposal envisaged malonyl-CoA as a metabolic switch to inhibit fatty acid oxidation in beta cells, thereby acting to modulate lipid signalling and amplify insulin secretion [4]. However, in other tissues this switching function largely depends on ACC2 [11], which is not present in beta cells [15, 16]. Our data suggest that ACC1 cannot subsume this role, and argue more generally against the metabolic switch hypothesis. Indeed, we observed no disinhibition of palmitate oxidation in the *Acaca* knockout model, nor did repletion of cellular fatty acid pools with exogenous palmitate revert the secretory defect. Likewise, the levels of key TAG and DAG species previously implicated in glucose-stimulated lipid turnover [32] were unaltered by *Acaca* gene deletion. This does not exclude the possibility that amplification factors might be derived from TAG [4] but rather that ACC1 (via metabolic switching and/or de novo fatty acid synthesis) does not contribute to the maintenance of the TAG precursor pool. Indeed, our results clearly decouple ACC1 from the GSIS amplification pathways and highlight the need for alternative mechanisms to explain the links between GSIS and de novo lipogenesis as exemplified here and in earlier studies using inhibition of ACC1 and/or FAS [15]. For example, ACC1 may influence membrane fluidity and vesicle fusion: mice with loss of liver kinase B1 (LKB1) in beta cells show disinhibition of ACC1, increased cellular fatty acid levels and enhanced membrane excitability and insulin secretion [42]. The reduction of some phospholipid species we observed with ACC1 loss might be consistent with these possibilities but this would need to be tested in future studies focusing on individual cellular compartments.

In contrast to the marked secretory defect observed in both our models, mice with deletion of the gene encoding FAS in *Ins2*-expressing tissues exhibited normal insulin secretion in vivo and ex vivo [35], suggesting that ACC1-mediated malonyl-CoA production or acetyl-CoA consumption, rather than subsequent FAS-mediated lipogenesis, may control beta cell function. This concept is consistent with the 30-fold augmentation of ACC activity in glucose-stimulated INS-1 cells, compared with only a threefold increase in FAS activity [17]. Therefore, it is plausible that changes in malonyl-CoA, rather than a product of downstream lipogenesis, directly couple the ACC1 pathway with insulin secretion, such as by altering protein malonylation [43].

Another possibility is that ACC1 controls insulin secretion by regulating cellular acetyl-CoA availability. Acetyl-CoA is a substrate of ACC1, and loss of ACC1/ACC2 activity in the liver dysregulates protein acetylation [20]. Moreover, beta cell acetyl-CoA is an important acute potentiator of insulin secretion, likely acting by enhancing protein acetylation [44]. However, in beta cells with persistent loss of ACC1 activity, as in our models, it is possible that chronically elevated acetyl-CoA levels may incur a secretory defect, analogous to the generation of glucotoxicity following long-term glucose exposure. For example, altered acetylation of proteins in the glycolytic pathway and tricarboxylic acid cycle, as in the ACC-null liver [20], may impair metabolic coupling in ACC1-null beta cells. Likewise, acetoacetate breakdown provides acetyl-CoA to ACC1 in beta cells via a pathway distinct from ATP citrate lyase [45]. It is therefore plausible that loss of ACC1 activity could result in persistent elevations of acetoacetate, contributing to defective beta cell function in βACC1KO and tmx-βACC1KO mice.

Our study has revealed, somewhat unexpectedly, that beta cell mass and individual beta cell size are reduced with lifelong loss of ACC1 activity in beta cells. This suggests a novel role for ACC1 in regulating beta cell growth during the marked expansion of beta cell mass that occurs during adolescence [28]. In contrast, beta cell mass was not significantly altered in tmx-βACC1KO mice 6 weeks post ACC1 deletion: this is perhaps not surprising, given that relatively little beta cell expansion is expected to occur during this adult period. Therefore, our results are consistent with ACC1 playing a role in the expansion, rather than the maintenance, of beta cell mass.

Consistent with our beta cell mass data, we observed decreased abundance of key translational/growth regulators in βACC1KO mice but not in tmx-βACC1KO mice. Interestingly, acetylation of P70S6K inhibits its kinase activity [46], suggesting a potential link between ACC1 activity, acetyl-CoA availability and the regulation of protein translation. Although total P70S6K levels decreased in proportion to the decrease in S6 phosphorylation in βACC1KO islets, a reduction in P70S6K activity associated with increased acetyl-CoA levels cannot be ruled out.

In summary, our study reveals a critical role for ACC1 activity in controlling insulin secretion and beta cell mass in vivo through mechanisms independent of neutral lipid abundance. We further propose that ACC1 activity is a positive regulator of protein synthesis and beta cell growth.

## Electronic supplementary material

ESM (PDF 924 kb)

## Data Availability

Individual data points are shown in the figures. Tabulated data are available upon request from the corresponding author.

## Abbreviations

ACC: Acetyl-CoA carboxylase
βACC1KO: Mouse with *Acaca* gene deletion in beta cells.
DAG: Diacylglycerol
DEXA: Dual energy x-ray absorptiometry
4EBP1: Eukaryotic translation initiation factor 4E binding protein 1
FAS: Fatty acid synthase
GSIS: Glucose-stimulated insulin secretion
HRP: Horseradish peroxidase
HSPA9: Heat shock protein 9
IPGTT: Intraperitoneal glucose tolerance test
KRBH: HEPES-buffered KRB
mTOR: Mechanistic target of rapamycin kinase
P70S6K: Ribosomal protein S6 kinase, polypeptide 1
TAG: Triacylglycerol
tmx-βACC1KO: Mouse with inducible *Acaca* gene deletion in beta cells
