# Supplementary material for: Disruption of beta cell acetyl-CoA carboxylase-1 in mice impairs insulin secretion and beta cell mass
Source: Diabetologia. 2018 Oct 17;62(1):99–111. doi: 10.1007/s00125-018-4743-7 (PMC6290731; doi:10.1007/s00125-018-4743-7)
Supplement: Supplementary file 1 — (PDF 924 kb) [file 125_2018_4743_MOESM1_ESM.pdf]

ESM Methods

Disruption of beta cell acetyl-CoA carboxylase-1 in mice impairs insulin secretion and beta cell mass

Mouse models

Experimental procedures were performed under guidance provided by the Garvan Institute/St.Vincent's Hospital Animal Ethics Committee (project 09/45) and the UK Home Office (project license 30/3146).

Acaca floxed mice (ACC1 flox) (loxP sites flanking exons 42 and 43 of the *Acaca* gene to facilitate deletion of the start of the carboxyltransferase domain and introduce a frame shift) have been described previously [1, 2]. *Acaca* floxed and *Ins2*cre mice were crossed to generate compound heterozygotes, which were crossed to generate cohorts of *Ins2*cre-*Acaca*$^{flox/flox}$ (βACC1KO) and littermate control *Ins2*cre (INS2cre) mice. INS2cre mice were used to control for the presence of the INS2cre transgene in all experiments for this line, as this transgene has been shown to cause glucose intolerance in some colonies [3], but not others [4], most likely due to aberrant human growth hormone mini-gene expression [5]. To enable tamoxifen-inducible disruption of ACC1 activity in beta cells, *Acaca* floxed mice were crossed with *Pdx1*creER mice (Tg(*Pdx1*-cre/*Esr1**)35.10Dam) [6] to generate compound heterozygotes, which underwent two more rounds of breeding to generate cohorts of experimental mice that received 2 x daily subcutaneous (interscapular) injections of 8mg tamoxifen in vegetable oil [7] at 10 weeks of age to generate tamoxifen-treated *Acaca*$^{flox/flox}$, *Pdx1*-creER (tmx-βACC1KO) and littermate control tamoxifen-treated *Acaca*$^{flox/flox}$ (tmx-Control) mice, providing temporal resolution on the role of ACC1 in beta cell function. Recombination of loxP sites was assessed by extraction of genomic DNA as previously described [8] and PCR performed using the primers TACAAACGCAAGAGTCATACTGG and CACACCAGTATTTGAATCAGCAA, REDTaq PCR mix (Sigma-Aldrich) and an annealing temperature of 60°C. PCR products were mixed with GelRed DNA stain (Biotium, Fremont, CA, USA), resolved using a 1.5% agarose gel and visualised with UV illumination. Each experimental cohort contained both control and knock-out mice, which were studied in a randomised order.

Mice were maintained on a C57Bl/6J background and were group housed in individually-ventilated cages within a barrier facility maintained at room temperature with 12 h light/dark cycles. Environmental enrichment was provided and mice had free access to a standard lab diet [9] and water.

Metabolic testing:

Glucose tolerance tests were performed between 8am and noon after a 16 h fast (with free access to water), by i.p. or i.v. injection of 20% glucose (2 or 1g/kg, respectively). Insulin action was assessed by i.p. injection of Actrapid insulin (0.75 U/kg, diluted in saline) at 2pm following a 6 h fast. Fed blood measurements were made at 9am (2 h after lights on). Blood sampling was performed from the tail. Mice were handled for injections before being returned to their home cages. Blood glucose was quantified by glucometer (Roche). Plasma was purified by centrifugation for hormone assays. Percentage body fat was determined by dual energy X-ray absorptiometry (DEXA) scan normalised to body weight.

Islet isolation, culture and insulin secretion assays:

Islet isolation, culture and insulin secretion assays were performed as previously described [10]. In brief, the pancreas of mice was perfused with a solution containing collagenase I,

collagenase II and thermolysin enzymes (Roche, Basel, Switzerland) via the common bile duct. The excised pancreas was digested at 37°C before mechanical disruption and purification of islets using a Ficoll-paque gradient (GE Healthcare, Chalfont St Giles, UK). Islets were washed and cultured overnight in RPMI1640 containing 11 mmol/l glucose and 10% FCS (ThermoFisher, Waltham, MA, USA). To assess ex vivo insulin secretion, islets were pre-incubated for 1 h in HEPES-buffered Krebs-Ringer buffer (KRBH) containing 0.1% BSA and 2 mmol/l D-glucose. Batches of five size-matched islets were incubated at 37°C for 1 h in KRBH/0.1% BSA supplemented with glucose, KCl (25mmol/l) or palmitate (0.4mmol/l palmitate coupled to 0.92% BSA [11]) as indicated.

Hormone and DNA assays:
Insulin was quantified by ELISA (Crystal Chem, Elk Grove Village, IL, USA) or RIA (Merck Millipore, Burlington, MS, USA). Glucagon was quantified by RIA (Merck Millipore). Total pancreatic hormone content was quantified following homogenisation in ice-cold acid–ethanol. Batches of islets were lysed and DNA content quantified by SYBR green assay (ThermoFisher, Waltham, MA, USA) using salmon-sperm DNA standards [11].

Islet metabolic studies:
Metabolic tracer measurements were made as previously described [12]. Batches of 100 islets were cultured for 2h at 37°C in KRBH containing 0.1% BSA and 2.8 or 20mmol/l D-glucose, with 0.5 x 10^6 or 0.07 x 10^6 MBq/mol ^{14}C[U]-glucose (respectively). Alternatively, batches of 100 islets were cultured for 2h at 37°C in KRBH containing 20mmol/l D-glucose and 0.4 mmol/l palmitate (coupled to 0.9% BSA), 0.8mmol/l L-carnitine and ^{14}C[U]-palmitate (1.85 x 10^6 MBq/mol). ^{14}C tracers were purchased from Perkin Elmer (Waltham, MA, USA). To quantify tracer oxidation, media was acidified and $^{14}CO_2$ trapped via reaction with 0.1 ml KOH and quantified by liquid scintillation spectrometry. To quantify tracer incorporation into lipids, a chloroform:methanol (2:1) extraction was performed, and the lipid fraction quantified by scintillation spectrometry.

Mass spectrometry (MS) based lipidomic analysis of islets was performed as previously described [13]. In brief, lipids were extracted from 100 islets per mouse using chloroform methanol (2:1, vol./vol.) with the following internal standards (Sigma-Aldrich): PC 13:0/13:0, PE (phosphatidylethanolamine) 17:0/17:0, PS (phosphatidylserine) 17:0/17:0, DAG (diacylglycerol) 15:0/15:0 and TAG (triacylglycerol) 17:0/17:0/17:0. Analysis was performed by electrospray ionisation-tandem MS using an API 4000 Q/TRAP mass spectrometer (Sciex, Framingham, MA, USA) with a turbo-ionspray source and Analyst 1.5 data system, following prior liquid chromatographic separation. Quantification of individual lipid species was performed using scheduled multiple-reaction monitoring in positive ion mode. Lipid concentrations were calculated by relating the peak area of each species to the peak area of the corresponding internal standard. PI species were related to the PE internal standard. For DAG and TAG species containing two or three of the same FA used as a neutral loss, the signal response was corrected by dividing by the number of copies of the fatty acid.

Histology:
Pancreases were excised, weighed, formalin fixed, paraffin-embedded, sectioned and immunostained for insulin (Sigma-Aldrich, St. Louis, MO, USA), glucagon (Abcam, Cambridge, UK) and/or Ki67 (Abcam), using Alexa Fluor conjugated secondary antibodies (ThermoFisher), and counterstained with DAPI [8]. For morphometry, stained sections were imaged with an Arperio slide scanner (Leica Biosystems, Wetzlar, Germany), and analysis

performed using Imagescope software (Leica Biosystems). For dual insulin/glucagon immunofluorescent staining, sections were imaged using a DMI6000 SP8 Confocal microscope (Leica Biosystems). β-cell mass was calculated as described [8] using data averaged from 3 pancreatic sections per mouse. β-cell size (mean individual β-cell volume) was estimated by dividing insulin-positive area (containing at least 1000 cells) by the number of cells/nuclei in that area, then converting to volume.

Western blotting:
Tissue/cells were lysed in RIPA buffer [14], protein quantified by BCA assay (ThermoFisher) and western blotting performed as previously described [10], using SDS-PAGE gels (with equal amounts of protein loaded per well) and Polyvinylidene difluoride (PVDF) membranes (ThermoFisher). Membranes were blocked and probed with the following antibodies, used in accordance with manufacturers guidelines: mTOR, phospho-P70S6K (Thr389), P70S6K, phospho-Ribosomal protein S6 (Ser235/236), phospho-Ribosomal protein S6 (S240/244), phospho-4EBP1 (Thr37/46), 4EBP1, β-Tubulin, and Horseradish peroxidase-conjugated secondary antibodies (Cell Signalling Technologies, Danvers, MA, USA); HSPA9 and 14-3-3b (Santa Cruz, TX, USA); Glucokinase (Abcam, Cambridge, UK). All primary antibodies were diluted 1 in 1000 before use except anti-HSPA9 which was diluted 1 in 500. All secondary antibodies were diluted 1 in 10,000. Chemiluminescent detection (GE Healthcare) was performed and band density was quantified using Image J software (NIH, Bethesda, MA, USA). ACC1 protein was identified by avidin-agarose bead pull-down, SDS-PAGE, transfer to PVDF, incubation with streptavidin-HRP (ThermoFisher) and chemiluminescent detection: this method utilises the affinity of the endogenous ACC1-bound biotin for avidin/streptavidin, and has been previously described [1].

Statistics:
Data are presented as mean ± SEM. Simple pairwise comparisons were made using two-tailed *t* tests (unpaired unless otherwise stated). Multiple comparisons were made using two-way ANOVA with Bonferroni post-tests. A p value <0.05 was regarded as statistically significant. Statistics were performed using Prism6 (Graphpad Software, San Diego, CA, USA).

References:

[1] Chow JD, Lawrence RT, Healy ME, et al. (2014) Genetic inhibition of hepatic acetyl-CoA carboxylase activity increases liver fat and alters global protein acetylation. Mol Metab 3: 419-431

[2] Lee J, Walsh MC, Hoehn KL, James DE, Wherry EJ, Choi Y (2014) Regulator of fatty acid metabolism, acetyl coenzyme a carboxylase 1, controls T cell immunity. Journal of immunology 192: 3190-3199

[3] Lee JY, Ristow M, Lin X, White MF, Magnuson MA, Hennighausen L (2006) RIP-Cre revisited, evidence for impairments of pancreatic beta-cell function. The Journal of biological chemistry 281: 2649-2653

[4] Fex M, Wierup N, Nitert MD, Ristow M, Mulder H (2007) Rat insulin promoter 2-Cre recombinase mice bred onto a pure C57BL/6J background exhibit unaltered glucose tolerance. The Journal of endocrinology 194: 551-555

[5] Brouwers B, de Faudeur G, Osipovich AB, et al. (2014) Impaired islet function in commonly used transgenic mouse lines due to human growth hormone minigene expression. Cell Metab 20: 979-990

[6] Gu G, Dubauskaite J, Melton DA (2002) Direct evidence for the pancreatic lineage: NGN3+ cells are islet progenitors and are distinct from duct progenitors. Development 129: 2447-2457
[7] Granot Z, Swisa A, Magenheim J, et al. (2009) LKB1 regulates pancreatic beta cell size, polarity, and function. Cell Metab 10: 296-308
[8] Cantley J, Choudhury AI, Asare-Anane H, et al. (2007) Pancreatic deletion of insulin receptor substrate 2 reduces beta and alpha cell mass and impairs glucose homeostasis in mice. Diabetologia 50: 1248-1256
[9] Wu LE, Samocha-Bonet D, Whitworth PT, et al. (2014) Identification of fatty acid binding protein 4 as an adipokine that regulates insulin secretion during obesity. Molecular Metabolism 3: 465-473
[10] Cantley J, Boslem E, Laybutt DR, et al. (2011) Deletion of protein kinase C delta in mice modulates stability of inflammatory genes and protects against cytokine-stimulated beta cell death in vitro and in vivo. Diabetologia 54: 380-389
[11] Cantley J, Burchfield JG, Pearson GL, Schmitz-Peiffer C, Leitges M, Biden TJ (2009) Deletion of PKCepsilon selectively enhances the amplifying pathways of glucose-stimulated insulin secretion via increased lipolysis in mouse beta-cells. Diabetes 58: 1826-1834
[12] Schmitz-Peiffer C, Laybutt DR, Burchfield JG, et al. (2007) Inhibition of PKCepsilon improves glucose-stimulated insulin secretion and reduces insulin clearance. Cell Metab 6: 320-328
[13] Boslem E, MacIntosh G, Preston AM, et al. (2011) A lipidomic screen of palmitate-treated MIN6 beta-cells links sphingolipid metabolites with endoplasmic reticulum (ER) stress and impaired protein trafficking. Biochem J 435: 267-276
[14] Li J, Cantley J, Burchfield J, et al. (2014) DOC2 isoforms play dual roles in insulin secretion and insulin-stimulated glucose uptake. Diabetologia 57: 2173-2182

ESM Figure 1

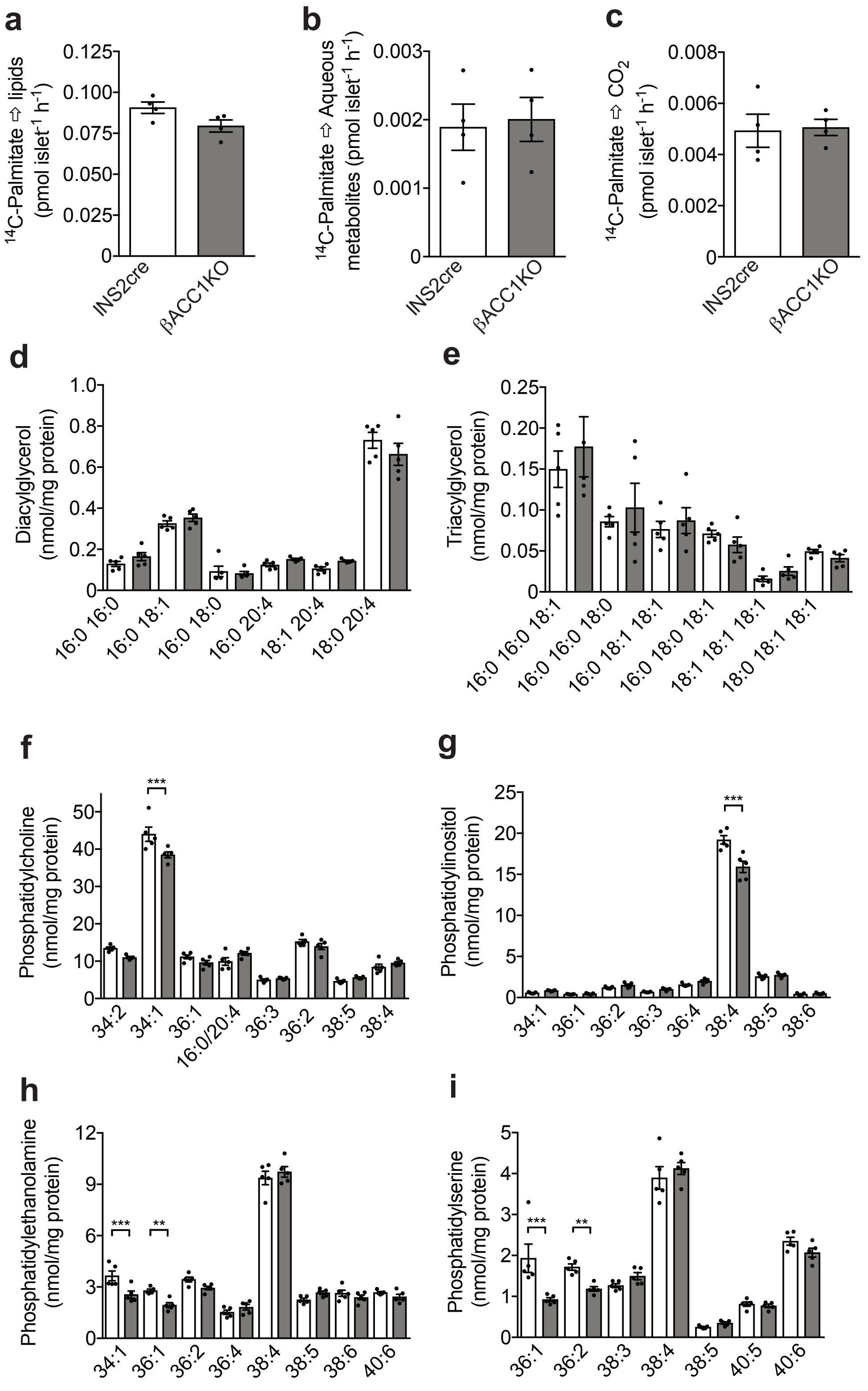

ESM Figure 1: Loss of ACC1 in beta cells does not alter lipid metabolism or neutral lipid abundance, but induces some phospholipid remodelling. A 14C[U]-palmitate tracer was used to quantify incorporation of exogenous fatty acids into cellular lipid pools (a), aqueous metabolites (b) and beta oxidation pathways (c). Mass spectroscopic lipidomic analysis of islet neutral lipid (d-e) and phospholipid (f-i) species. Key: White bars, INS2cre; grey bars, βACC1KO. Sample sizes: (a-c) n=4 batches of islets from independent mice. (d-i) n=5 batches of islets from independent mice. Statistical analysis: unpaired 2-tailed t-tests; p<0.01**, p<0.001***. Data are presented as mean ±SEM.

ESM Figure 2

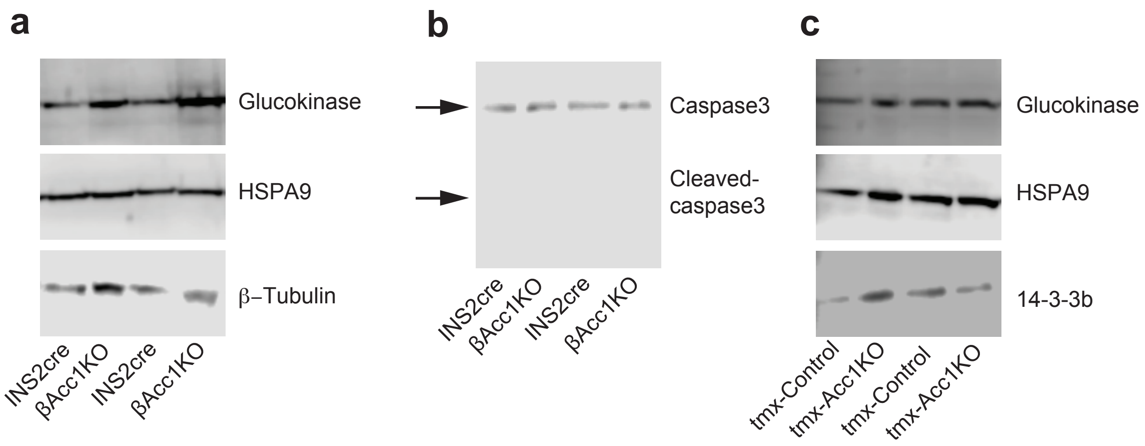

ESM Figure 2: Abundance of proteins involved in glucose sensing and apoptosis are not defective in ACC1 null islets. Western blotting of islet lysates using antibodies against the indicated proteins, using islets isolated from βACC1KO and INS2cre mice (a, b), or tmx-βACC1KO and tmx-Control mice (c). Arrows on capsase 3 blot (b) indicate expected location of full length (35kDa, upper arrow) and cleaved (17kDa, lower arrow) forms of the protein. Sample sizes: each lane represents islets from an independent mouse.
